# CLOCK 3111T/C Variant Correlates with Motor Fluctuation and Sleep Disorders in Chinese Patients with Parkinson's Disease

**DOI:** 10.1155/2018/4670380

**Published:** 2018-02-04

**Authors:** Fan Lou, Ming Li, Xiaoguang Luo, Yan Ren

**Affiliations:** ^1^Department of Neurology, First Affiliated Hospital of China Medical University, Shenyang, Liaoning, China; ^2^Department of Neurology, The Fourth Affiliated Hospital of China Medical University, Shenyang, Liaoning, China

## Abstract

**Background:**

The clock genes controlling biological rhythm play an important role in the pathophysiology of aging. The purpose of this study was to determine whether there is an association between a variant of the circadian locomotor output cycles kaput (CLOCK) gene and circadian dysfunction of Parkinson's disease (PD).

**Methods:**

Six hundred and forty-six cases of Parkinson's disease from consecutive outpatients and inpatients ward from our hospital were included in this study. Kompetitive allele-specific PCR was used to determine the frequency distribution of genotypes and alleles. The examinations for the PD group were assessed in person in order to evaluate motor symptoms, cognitive function, sleep, and depression, including the Unified Parkinson's Disease Rating Scale (UPDRS), Mini-Mental State Examination (MMSE), Pittsburgh Sleep Quality Index (PSQI), and 17-item Hamilton Rating Scale for Depression (HAMD-17).

**Results:**

Motor fluctuation (*P* < 0.001) and sleep disorders (*P*=0.007) were significantly different between the two groups. These correlations persisted after adjusting for confounding risk factors by further binary logistic regression analysis, suggesting that the CLOCK 3111T/C variant was associated with motor fluctuation (OR = 1.080, *P* < 0.001) and a subjective sleep disorder (OR = 1.130, *P*=0.037).

**Conclusion:**

The CLOCK 3111T/C variant can be an independent risk factor for motor fluctuation and sleep disorder in Parkinson's disease.

## 1. Background

Parkinson's disease (PD) is a degenerative neurological disorder characterized by the loss of dopaminergic neurons in the substantia nigra. It is generally believed that the interaction between multiple genes and environmental factors contributes to an extremely complicated mechanism of PD [1]. Symptoms of circadian dysfunction are the typical symptoms of neurodegenerative diseases, such as Alzheimer's disease (AD) and PD [2–4]. Impaired circadian function in PD is implicated not only in impaired sleep and alertness but also in autonomic, behavior, mood, and fluctuating motor manifestations [5]. The daily life of patients with PD can be beset by circadian dysfunction, which may be more severe than motor symptoms alone and can become a heavy burden on the family.

The biological rhythm of the human body is regulated by the circadian system. Many physical activities are controlled by the circadian clock, such as the sleep-awake cycle, blood pressure, heart rate, respiration, temperature, visceral activity, immunity, neuroendocrine activity, cell division, and even DNA function. Recently, the clock genes that regulate the circadian rhythm in both the central and peripheral oscillators, located in the suprachiasmatic nucleus, including CLOCK (circadian locomotor output cycles kaput), BMAL1, Period (Per), and Cryptochrome (Cry) genes, have aroused the interest of many researchers [6]. Among those genes, CLOCK is the key gene that regulates the circadian rhythm in the brain and periphery, which can modulate the dopamine synthesis [7]. The 3111T/C is located in the 3′-untranslated region of the CLOCK gene, with a T→C transversion, which has previously been found to be associated with psychobehavioral control, hormone secretion, mood, and sleep [8, 9]. Recently, polymorphisms of some related clock genes have been reported to be associated with the circadian rhythm disturbances of PD [10, 11]. In a previous study, a significant association with CLOCK 3111T/C in the dominant model in PD was observed, when compared to that of the controls. This suggests that the circadian rhythm gene can be considered to promote the pathophysiology of PD [12]. However, current knowledge regarding the relationship between circadian dysfunction in PD and the CLOCK gene variant is scarce.

Studies on the relationship between a single-nucleotide polymorphism of clock genes and circadian dysfunctions of PD may provide more insight into the genetics of motor and nonmotor symptoms of PD. Therefore, in the present study, we examined the association between a polymorphism of the CLOCK gene with symptoms of circadian dysfunction in PD in a northeastern Chinese population using a retrospective case study.

## 2. Methods

### 2.1. Subjects

The present study included 646 patients with PD who were from an unrelated Chinese population in the northeastern provinces of China. The patients were recruited from the movement disorder clinic and inpatient ward of the Department of Neurology, the First Affiliated Hospital of China Medical University, Shenyang, China, from May 2010 to October 2015. The diagnosis of PD was made based on the UK PD Brain Bank Criteria by movement disorder neurologists [13]. Ages of the all participants ranged from 36 to 88 years. All patients underwent the Mini-Mental State Examination (MMSE). We excluded those whose score fell below 20 for individuals who received 6 years of schooling or less (primary school education) and below 24 for individuals who received more than 6 years of schooling (junior and above). Patients with Parkinsonism-plus and those who worked shift work were also excluded. All patients provided their written informed consent. The Ethics Committee of China Medical University approved the study. Demographic data were collected for all patients with PD.

### 2.2. Evaluation

We completed the following standard assessment measures when patients had not taken levodopa or other PD medications for at least 12 h (the “off state”):

1. Hoehn and Yahr staging was used for determining the stage of the disease.
2. Severity of Parkinsonism was evaluated using the Unified Parkinson's Disease Rating Scale (UPDRS) II + III, and the tremor, postural, and gait instability scores were further calculated according to the report from Kang et al. [14].
3. Depression was determined by a score ≥8 on the 17-item Hamilton Rating Scale for Depression (HAMD-17).
4. The motor fluctuation of the PD group was determined by a score of >1 on item 39 from the UPDRS IV [15].
5. A subjective sleep disorder in the PD group was determined by a Pittsburgh Sleep Quality Index (PSQI) ≥ 7 [16].
6. Orthostatic hypotension was determined by a decrease in the systolic blood pressure > 20 mmHg or a decrease in the diastolic pressure > 10 mmHg, accompanied by the symptoms of hypoperfusion, such as dizziness, within 3 minutes of moving into the upright position.
7. Dyskinesia was determined using the total score of items 32 to 35 from the UPDRS IV.

### 2.3. DNA Extraction and Genotyping

Genomic DNA was extracted from leukocytes using the sodium dodecyl sulfate-proteinase K phenol-chloroform method. DNA purity was assessed by spectrophotometry, and DNA samples were stored at −20°C. Genotypes were determined using the Kompetitive allele-specific PCR (KASP) assay [17]. Each amplification reaction contained 50 ng/*μ*l of template DNA, KASP 2x Master Mix standard ROX (LGC Genomics, UK), and KASP-by-Design assay mix (LGC Genomics, UK). For the allele-specific amplification of CLOCK gene 3111T/C, the primers (including forward primers marked by FAM and HEX) were 5′GAAGGTGACCAAGTTCATGCTAAAACACTGTCAGAACTGGCTG3′ and 5′GAAGGTCGGAGTCAACGGATTCCTAAAACACTGTCAGAACTGGCTA3′. During PCR, the thermocycling conditions of all the primers were 15 min at 94°C, followed by 10 cycles of 94°C for 20 s and 61°C for 1 min (dropping −0.6°C per cycle to achieve a 55°C annealing temperature), followed by 26 cycles of 94°C for 20 s and 55°C for 1 min. After amplification, PCR plates were read with a SpectraMax M5 FRET capable plate reader (LGC Genomics, UK) using the recommended excitation and emission values. Data were subsequently analyzed using KlusterCaller software (LGC Genomics, UK) to identify single-nucleotide polymorphism genotypes.

### 2.4. Statistical Analysis

The continuous variables are presented as mean ± SD and categorical variables as percentages. Differences of the continuous variables were evaluated by the Student's *t*-test, depending on the shape of the distribution curves. Categorical variables were compared using the *χ*^2^ test. All genotype frequencies were checked using the Hardy-Weinberg analysis in two groups through the *χ*^2^ test [18]. Differences in the distributions of the alleles and genotypes between the cases and controls were analyzed using the *χ*^2^ test. The association between the CLOCK gene polymorphisms and circadian dysfunctions of PD was estimated by computing the odds ratios (ORs) and 95% confidence intervals (CIs) from a binary logistic regression analysis using the backward-stepwise method. The statistical analyses were performed using SPSS version 20.0 (IBM, USA) with the limit of significance set at *P* < 0.05 (two-tailed).

## 3. Results

We identified the CLOCK T3111C variant in 124 of 646 patients with PD, including the TC and CC genotypes, in a northeastern Chinese population, and we found a significant difference between patients with PD and the healthy controls in the dominant model [12]. Therefore, in order to further explore the clinical characteristics of the patients with variant, we divided the patients into the following two groups: T3111C variant carriers (TC and CC genotypes) and noncarriers (TT genotype). We compared the 124 carriers and 522 noncarrier patients using a complete set of clinical data. The demographic data and clinical profiles of the 3111T/C carriers and noncarriers are shown in Table 1. There were no significant differences in age, sex, course of diseases, years of education, Hoehn-Yahr stage, or severity of motor symptoms (UPDRS II + III) between the carriers and noncarriers (*P* > 0.05), indicating that the two groups were comparable.

As shown in Table 1, our study showed that the 3111T/C variant carriers scored significantly higher in motor fluctuation subitems than the noncarriers (1.93 ± 1.88 versus 0.83 ± 1.41, *P* < 0.001) did and also had more subjective sleep disorders (38.7% versus 26.4%, *P*=0.007). We defined the onset age as the age at which the initial symptom appeared in confirmed patients. We defined early-onset PD (EOPD) as patients whose onset age was ≦50 years. The carriers had a significantly higher frequency of EOPD than the noncarriers (9.7% versus 5.0%, *P*=0.046). However, no differences in dyskinesia, depression, or orthostatic hypotension were found between the carriers and the noncarriers (*P* > 0.05).

In order to further confirm the association between the gene variant and motor fluctuation and exclude other potential factors, binary logistic regression was used to examine the relationship. As shown in Table 2, after adjusting for age, disease duration, Hoehn–Yahr stage, impact of levodopa therapy, and EOPD, three variables, including the 3111T/C variant (*P* < 0.001), UPDRS II + III (*P* < 0.001), and Hoehn-Yahr stage (*P*=0.044), were significantly associated with motor fluctuation in the logistic regression. These results showed that a polymorphism of CLOCK 3111T/C can be an independent risk factor for motor fluctuation (OR = 1.080, 95% CI = 1.057–1.104, *P* < 0.001). Similarly, as illustrated in Table 3, subjective sleep disorder was associated with a polymorphism of CLOCK 3111T/C (*P*=0.037), UPDRS II + III (*P* < 0.001), and depression (*P* < 0.001). This showed that a polymorphism of CLOCK 3111T/C can be an independent risk factor for the sleep disorder in Parkinson's disease (OR = 1.130, 95% CI = 1.007–1.2689, *P*=0.037).

## 4. Discussion

PD is multifactorial with many features exhibiting diurnal fluctuations, such as thermoregulation disorders, circadian variation of blood pressure and heart rate, emotional dysregulation, and even reactions to anti-PD pharmacotherapy that manifest as abnormalities in rhythm [3, 19–23]. Particularly, motor activity can vary throughout the day [24]. Recently, increasing studies show that these circadian rhythm dysfunctions of neurodegenerative disorders are associated with the abnormal regulation of clock genes [2, 4].

In the current study, we examined the association of a polymorphism of CLOCK gene with symptoms of circadian dysfunction in PD in a northeastern Chinese population using a retrospective case study. We found that 3111T/C variant carriers scored significantly higher than noncarriers in motor fluctuation, a subitem of UPDRS. Normal motor activity gradually increased during the daytime and reached the lowest level at night. However, the rhythm is modified in patients with PD, and motor fluctuation is very common [25]. Motor fluctuation is considered to be associated with a longer disease progression and higher levodopa daily dose [26]. In our research, there were no differences in the initial dosage of levodopa and the levodopa therapy duration between the two groups (*P* > 0.05), which is consistent with the results from other studies [20, 27]. Therefore, we inferred that the main influencing factors of motor fluctuation could be attributed to the gene variant rather than the long-term effects of levodopa. In order to exclude the impact of related factors, we performed a logistical analysis. After adjusting for probable risk factors, such as age, sex, disease duration, severity of disease, and impact of levodopa therapy, motor fluctuation was still related to the 3111T/C variant and the risk of motor fluctuation of carriers was 1.077 times that of noncarriers, which shows that motor fluctuation may be associated with the variant of the CLOCK gene in the circadian system. Many studies found that fluctuations in motor activity may be caused by the circadian system. Boulamery found that rhythmic changes induced by lesions in unilateral 6-OHDA PD animal model, which has a loss of circadian rhythmicity, had reduced locomotor activity [28]. Other studies found that exposure to light or administration of the melatonin receptor antagonist facilitates recovery of motor function in chronic experimental models of PD [29, 30]. There is increasing evidence for the existence of interactions between the circadian rhythm and dopamine system and the modulation of dopamine synthesis by circadian genes. The CLOCK gene can regulate the transcription of tyrosine hydroxylase [31], which is the rate-limiting enzyme for dopamine synthesis. CLOCK can also affect the dopaminergic activity at the posttranscriptional level, which leads to the motor fluctuations [32]. Above all, our results indicate that patients with PD who are CLOCK 3111T/C variant carriers are more susceptible to the development of motor fluctuation than their wild-type counterparts.

Our study also found that since the 3111T/C variant carriers scored significantly higher than the noncarriers on the PQSI, carriers were associated with a higher risk of subjective sleep disorder when compared to that of the noncarriers. Numerous studies show that the polymorphism of CLOCK 3111T/C is associated with sleep disturbance in human beings, which could affect diurnal behavior pattern [33, 34]. Disturbed sleep-wake cycles are common nonmotor manifestations of PD, affecting up to 90% of the patients. It is suggested that the etiology of sleep disorders in PD depends on the adverse effects of antiparkinsonian medications and primary neurodegeneration of central sleep regulatory areas [35, 36]. We also found that patients with PD who have the CLOCK gene variant are more susceptible to the development of sleep disorders after adjusting for impact factors related to sleep, such as depression, severity of motor symptoms, age, sex, and disease duration. Therefore, we further confirmed that the polymorphism of CLOCK 3111T/C can be an independent risk factor for sleep disorders in PD. We also found that the proportion of patients with EOPD in the carrier group was significantly higher than that in the noncarrier group. This may reveal that the 3111T/C variant may play a role in lowering the onset age of PD and needs to be confirmed by further research.

In previous studies, different researchers have found that the polymorphism of CLOCK 3111T/C is associated with psychiatric disorders [37, 38]. Hua et al. [10] found that a polymorphism of Tef, which is a downstream regulatory gene of CLOCK, is associated with the depression of PD in Chinese population. Although in our study we found no statistical significant difference between the two groups in depression, a tendency could be found, which warrants further research. However, with regard to the other symptoms of circadian rhythm disorders, such as dyskinesia, orthostatic hypotension, and response to levodopa, no differences were found between the two groups. Different results may be attributed to the different loci of clock genes, sample size, different regions and nations, and different statistical methods.

Although the circadian timing controls the temporal patterning of molecular, cellular, and physiological processes throughout the body and the potential disruption of this timing system in PD would be expected to produce widespread symptoms [6], there is little known about the mechanism of the variant of clock genes leading to circadian dysfunctions in PD. However, pharmacotherapeutics and behavior pattern can intervene with pathological changes caused by circadian rhythm dysfunction [39]. Daily evening exposure to supplemental bright light improved sleep quality and mood, as well as bradykinesia and rigidity [40].

In conclusion, our results showed that the 3111T/C variant of CLOCK gene was closely related to the occurrence of motor fluctuation and sleep disorders in a northeastern Chinese population. It raises the possibility that circadian dysfunctions experienced by patients with PD may not merely be a subsidiary of the motor symptoms but an integral part of circadian system related to the disease itself. Our study confirmed a correlation between the symptoms of the circadian dysfunctions and Parkinson's disease from the view of the gene level. These findings suggest that circadian system may be a novel diagnostic and therapeutic target in motor fluctuation and sleep disorders with PD. However, multiple factors should be involved, such as genetic background, gene-environment interactions, and interaction of other clock genes. Therefore, more research is required to better understand the relationship between the polymorphism of clock genes and clinical profiles of PD.

## 5. Conclusion

CLOCK 3111T/C variant can be an independent risk factor for motor fluctuation and sleep disorder in Parkinson's disease in Chinese population.

## Figures and Tables

**Table 1:** Demographic data and clinical profiles of carriers and noncarriers.

	CLOCK 3111T/C carriers (TC + CC)	CLOCK 3111T/C noncarriers (TT)	*P* value
	*n* = 124	*n* = 522	
Age (years)	62.35 ± 10.19	61.70 ± 10.13	0.515
Sex (%)	53 (42.7)	263 (50.4)	0.126
Onset age (years)	58.52 ± 10.33	58.02 ± 10.79	0.645
EOPD (%)	12 (9.7)	26 (5.0)	0.046^a^
Disease duration (years)	3.90 ± 3.18	3.74 ± 3.04	0.550
Family history (%)	7 (5.6)	31 (5.9)	0.987
Formal education (years)	9.47 ± 3.38	9.63 ± 3.65	0.657
Hoehn-Yahr stage	1.84 ± 0.78	1.74 ± 0.73	0.165
UPDRS II + III	41.81 ± 19.73	40.26 ± 19.04	0.417
Mean tremor score	4.27 ± 4.19	4.00 ± 3.79	0.476
Mean PIGD score	7.71 ± 5.26	8.02 ± 5.25	0.691
Motor fluctuation	1.93 ± 1.88	0.83 ± 1.41	0.000^b^
Dyskinesia	0.33 ± 0.94	0.25 ± 1.03	0.453
L-dopa therapy initial dosage (mg/day)	148.7 ± 38.86	154.6 ± 41.12	0.215
L-dopa therapy duration (year)	3.22 ± 2.57	2.95 ± 2.80	0.071
Good response to levodopa	107 (86.3)	474 (90.8)	0.133
MMSE	25.23 ± 3.51	25.37 ± 3.32	0.682
Sleep disorder (%)	48 (38.7)	138 (26.4)	0.007^a^
Depression	10.26 ± 3.67	8.88 ± 3.73	0.183
Orthostatic hypotension (%)	16 (12.9)	69 (13.2)	0.926

The *χ*^2^ square test was used to compare the categorical variables. Data are expressed as numbers with percentages in parentheses or as mean ± SE. UPDRS: Unified Parkinson's Disease Rating Scale; MMSE: Mini-Mental State Examination; EOPD: early-onset PD; PIGD: postural instability and gait difficulty. ^a^*P* < 0.05 and ^b^*P* < 0.01.

**Table 2:** Binary logistic regression of association between motor fluctuation and CLOCK 3111T/C variant.

Variable	OR	95% CI	*P* value
3111T/C variant	1.080	1.057–1.104	0.000^a^
Age (years)	0.645	0.202–2.067	0.461
Gender	0.962	0.649–1.424	0.845
Disease duration (years)	1.656	0.508–5.395	0.403
Hoehn-Yahr stage	0.940	0.885–0.998	0.044^b^
UPDRS II + III	4.698	2.868–7.695	0.000^a^
L-dopa therapy duration (year)	0.884	0.885–0.998	0.440
L-dopa therapy initial dosage (mg/day)	1.179	0.961–1.558	0.115
Response to levodopa	0.884	0.780–1.002	0.053
Mean PIGD score	1.002	0.945–1.062	0.957
EOPD	0.911	0.662–1.252	0.565

UPDRS: Unified Parkinson's Disease Rating Scale; MMSE: Mini-Mental State Examination; EOPD: early-onset PD. ^a^*P* < 0.01 and ^b^*P* < 0.05.

**Table 3:** Binary logistic regression of association between sleep disorders and CLOCK 3111T/C variant.

Variable	OR	95% CI	*P* value
3111T/C variant	1.130	1.007–1.268	0.037^a^
Age (years)	1.008	0.989–1.028	0.407
Gender	1.348	0.905–2.008	0.142
Disease duration (years)	1.039	0.971–1.111	0.266
Hoehn-Yahr stage	1.098	0.795–1.516	0.571
UPDRS II + III	1.044	1.022–1.066	0.000^b^
Mean PIGD score	0.976	0.917–1.038	0.443
Depression	3.872	2.455–6.135	0.000^b^
Response to levodopa	1.044	0.986–1.106	0.141

UPDRS: Unified Parkinson's Disease Rating Scale; MMSE: Mini-Mental State Examination. ^a^*P* < 0.05 and ^b^*P* < 0.01.
